# Not just conspiracy theories: Vaccine opponents and proponents add to the COVID-19 ‘infodemic’ on Twitter

**DOI:** 10.37016/mr-2020-38

**Published:** 2020-09-09

**Authors:** Amelia M. Jamison, David A. Broniatowski, Mark Dredze, Anu Sangraula, Michael C. Smith, Sandra C. Quinn

**Affiliations:** (1) Center for Health Equity, University of Maryland, USA; (2) Institute for Data, Democracy, and Politics & Department of Engineering, Management and Systems Engineering, The George Washington University, USA; (3) Department of Computer Science, Johns Hopkins University, USA; (4) Department of Engineering, Management and Systems Engineering, The George Washington University, USA; (5) Department of Family Science & Center for Health Equity, University of Maryland, USA

## Abstract

In February 2020, the World Health Organization announced an ‘infodemic’ -- a deluge of both accurate and inaccurate health information -- that accompanied the global pandemic of COVID-19 as a major challenge to effective health communication. We assessed content from the most active vaccine accounts on Twitter to understand how existing online communities contributed to the ‘infodemic’ during the early stages of the pandemic. While we expected vaccine opponents to share misleading information about COVID-19, we also found vaccine proponents were not immune to spreading less reliable claims. In both groups, the single largest topic of discussion consisted of narratives comparing COVID-19 to other diseases like seasonal influenza, often downplaying the severity of the novel coronavirus. When considering the scope of the ‘infodemic,’ researchers and health communicators must move beyond focusing on known bad actors and the most egregious types of misinformation to scrutinize the full spectrum of information -- from both reliable and unreliable sources -- that the public is likely to encounter online.

## Implications

On February 2, 2020, the World Health Organization declared that the spread of the novel coronavirus, nCov-2019, was accompanied by an ‘infodemic’ described as “an overabundance of information -- some accurate and some not” that was inhibiting the spread of trustworthy and reliable information (World Health Organization, 2020). Even under the best circumstances, effective health communication during the early stages of a pandemic is challenging, as it can be difficult to communicate high levels of scientific uncertainty to the public and information is constantly changing (Vaughan & Tinker, 2009). With the ‘infodemic,’ health information is competing against a veritable “tsunami” of competing claims, many of which are amplified across social media with greater speed and reach (Zarocostas, 2020). While much of the ensuing media attention has focused narrowly on misinformation, an ‘infodemic’ is characterized by volume, not by the quality of information. Online health misinformation, defined as “a health-related claim of fact that is currently false due to a lack of scientific evidence” (Chou et al., 2018), was a growing issue prior to the pandemic, but the scope of the problem has increased dramatically with the spread of COVID-19.

Misinformation is commonly attributed to sources with low journalistic integrity and, therefore, low credibility (e.g., Grinberg et al., 2019). These sources typically promote conspiracy theories and other fringe opinions. In this study, we argue that even high-credibility sources emphasizing factual and accurate reporting can be vectors of misinformation when faced with a highly uncertain context (see also, Reyna, 2020). It is important to recognize the full spectrum of misinformation -- from false facts, to misleading use of data, unverifiable rumors, and fully developed conspiracy theories -- as all types can impede effective communication.

In this analysis, we focused on communication within existing vaccine-focused communities on Twitter. Recent studies have demonstrated that messages pertaining to vaccination are one of the most active vectors for the spread of health misinformation and disinformation (Broniatowski et al., 2018; Larson, 2018; Walter et al., 2020). Although a small fraction of the general public, vaccine opponents have an outsized presence online and especially on Twitter (Pew Research Center, 2017). Anti-vaccine arguments have also been amplified by known malicious actors including bots and state sponsored trolls (Broniatowski et al., 2018). Despite Twitter’s recent efforts to limit the spread of misleading and false health claims, many vaccine opponent accounts remain active on the platform. As news of a novel coronavirus outbreak in Wuhan, China started to spread in the American media, accounts that frequently tweet about vaccines -- both those in favor and those opposed -- were among the first to start regularly tweeting about the novel virus and the public health community’s reaction.

It is also important to recognize the diversity of viewpoints within the broader Twitter vaccine community. Although some accounts tweet almost exclusively about vaccines, many others also discuss other types of content, making it possible to identify subgroups based on shared interests such as politics, public health, or current events. To better understand the spread of online health misinformation, and potentially mitigate its impact, we believe it is necessary to first understand the communication norms and topics of conversation that shape these varied subgroups. Qualitative research has demonstrated that vaccine-hesitant attitudes likely serve as a signal of social identity and can form a type of social capital in community development (Attwell & Smith, 2017). We believe a similar function may help explain the emergence of online subgroups with shared interests and communication norms, for both vaccine opponents and proponents. While a great deal of research has focused on “anti-vaxxers” online (i.e., vaccine opponents), less has studied their pro-vaccine counterparts, many of whom use Twitter as a platform not to promote vaccines directly, but to debunk and refute claims made by vaccine opponents.

To date, the majority of news coverage on COVID-19 misinformation has tended to focus on unmistakably false claims including conspiracy theories, unchecked rumors, false prevention methods, and dubious “cures” (see also Liu et al., 2020). Many of these claims have been promulgated by vaccine opponents. This should not be a surprise; many of these accounts are adept at quickly adapting new information to fit it into existing narratives that align with worldviews. Most recently this took the form of criticizing US public health measures to limit the disease spread -- from launching the #FireFauci hashtag calling for Dr. Anthony Fauci’s dismissal from the President’s COVID-19 Response Team (Nguyen, 2020), to the dissemination of once-fringe conspiracy theories targeting Bill Gates and 5G wireless to more mainstream audiences (Wakabayashi et al., 2020), to appearing as a prominent presence in the multiple “resistance” protests held in state capitals to protest social distancing measures (Wilson, 2020). Most egregious was the Plandemic propaganda undermining and discrediting research on a future COVID-19 vaccine (Zadrozny & Collins, 2020). These instances also suggest an increasing overlap between the altright and vaccine opposition in the United States (Frenkel et al., 2020; Zhou et al., 2019). Although this type of highly visible misinformation can be dangerous, it is often easily debunked and may primarily appeal to fringe audiences. We wanted to know what vaccine opponents were sharing, beyond conspiracy theories, and whether topics varied by subgroup. We found that while roughly ⅓ of topics were likely misinformation, another ⅓ were discussion topics, and slightly under ⅓ relied on more reliable information sources, including public health announcements.

We found that vaccine advocates, and especially those who did not have medical, scientific, or public health expertise (i.e., they were not doctors, researchers, or affiliated with health organizations), but were primarily engaging in debates with vaccine opponents, have promoted mixed messages, some of which overlapped with vaccine opponents’ messages downplaying the severity of the pandemic. This is where the nuances of types of misinformation are important -- tweeting a fact misrepresenting disease severity is not the same as tweeting a conspiracy theory that suggests vaccines are a government plot to track citizens -- they are both part of what makes an ‘infodemic’ so challenging. COVID-19 has proven to be both highly contagious and deadly, a combination that makes the current pandemic particularly challenging to contain (CDC COVID-19 Response Team et al., 2020). By focusing on the greater risks of influenza and urging Americans to get an influenza vaccine, early messaging on COVID-19’s severity was not clearly communicated. In late May, a Gallup Poll found that while roughly two-thirds of Americans believed that coronavirus was deadlier than seasonal flu, there were still a significant number who persisted in believing the virus was less deadly including a majority of Republicans (Ritter, 2020). Singh et al. found “Flu Comparisons” to be among the most prevalent myths on Twitter early in the COVID-19 pandemic (2020). Recent work suggests that exposure to misinformation on social media -- including messages downplaying disease severity -- was associated with a lower likelihood of individuals engaging in social distancing practices (Bridgman et al., 2020).

What does this mean for efforts to address the COVID-19 ‘infodemic’ on Twitter? First, we need to know which accounts are spreading misinformation. To the best of our knowledge, most these highly active vaccine accounts are genuine users -- not bots. While recent reports suggest that up to 50% of accounts tweeting about COVID-19 could be likely bots, our findings highlight the role of real people disseminating information within existing online communities (Roberts, 2020). Limiting fully automated accounts and misuse of amplification tools is still useful to weed out the most egregious violations but is unlikely to be helpful in this specific context. Fact checking content may be more effective, but may still not capture more nuanced forms of misinformation, especially in discussion-based topics. Online communities are not homogenous; our findings suggest online rumors tend to circulate within smaller subgroups. While many topics overlap, specific arguments are likely to vary. This suggests that a one-sizefits-all approach to combating misinformation is unlikely to work on both vaccine-oriented vaccine opponents and vaccine opponents that are motivated by conservative politics. Similarly, while health organizations and health professionals may be sharing reliable information on Twitter, some of their well-meaning allies, especially the self-proclaimed vaccine activists, may inadvertently be sending out conflicting health information. Staying aware of this subgroup of proponents and developing easily shared evidence-based online resources may be one way to improve the quality of information being shared.

Second, we need to recognize that while the most obvious sources of misinformation (for instance, known anti-vaccine and conspiracy theory accounts) pre-COVID-19 will continue to spread misinformation during the pandemic, even trusted and reliable sources can still contribute to the ‘infodemic’ by spreading falsehoods and misleading facts. Further, given that these sources may be more trusted by the public, these claims are less easily debunked or dismissed. An ‘infodemic’ consists of information from multiple sources: the scientific community, policy and practice, the media, and social media (Eysenbach, 2020). As information is translated between sources, distortions can occur particularly as complex science is reinterpreted by lay audiences. As the most accessible and least filtered source, social media is likely to be most vulnerable to misinformation. By focusing only on the most blatant forms of misinformation and the actors sharing them, both the media and scholars may inadvertently mislead the public into believing that they haven’t been exposed to misinformation about COVID-19, or that this egregious forms of misinformation are the most common. By drawing attention to fringe theories, well-meaning users may also further amplify misinformation (Ahmed et al., 2020). This is why we urge scholars of misinformation to go beyond the most visible forms of misinformation to highlight the complexities and subtleties that make an ‘infodemic’ so challenging.

## Findings

### Finding 1: A plurality of top accounts oppose vaccination, and likely represent human users.

Of the 2,000 accounts in our sample, 45% (*n*=905) opposed vaccination, 24% (*n=*479) were in favor of vaccination, 15% (*n=*311) were no longer publicly available on Twitter, and 15% (*n*=305) did not indicate a clear position on vaccines. Among accounts that were retweeted at least once, vaccine opponents were retweeted significantly more frequently than vaccine proponents, *t(1210)=6.86, p<0.001*, after applying a logarithmic transform to correct for skewed data.

Using Botometer (Davis et al., 2016), the majority of accounts had low scores (< 0.2), indicating a low likelihood of automation. After applying a logistic transform to control for floor and ceiling effects, we did not detect any significant difference in bot scores between vaccine opponent and proponent accounts, *t(1368) = 0.79, p=0.43*. Pew Research Center uses a threshold of 0.43 to determine likely bots (Wojcik et al., 2018). Applying this criterion, of the 1,754 accounts with complete data, 17% (*n=*298) were likely to be bots. Additionally, 12% of all accounts (*n*=246) did not have available bot scores, suggesting the accounts had switched privacy settings (n=198, 10%) or had been closed (n=48, 2%) either voluntarily or because they had been removed and purged from Twitter for violation of terms of service (Twitter Help Center, n.d.).

### Finding 2: A significant proportion of both opponents and proponents’ tweet primarily about vaccines. Vaccine opponent subgroups emerged around conservative politics and conspiracy theories. Vaccine proponent subgroups emerged around doctors and researchers and health organizations.

Roughly a quarter of all accounts (*n*=498; 25%) posted primarily vaccine content. These dedicated accounts disproportionately opposed vaccination (oppose *n=* 398, 80%, favor *n*=100, 20%), (*X*^*2*^ = 72.56, p<0.001). For vaccine opponents, vaccine-focused accounts are those that post almost entirely vaccine related content with few posts about other topics. This is one of the more cohesive communities we observed, with accounts retweeting information disseminated from a handful of anti-vaccine activists. For vaccine proponents, vaccine-focused accounts represent a combination of health advocates who tweet almost solely about vaccines but also accounts we dubbed “*anti* anti-vax” who use Twitter to engage in debates with anti-vaccine accounts and to debunk or mock anti-vaccine arguments. Another 347 (17%) accounts were sharing “mixed content” not easily classified into a single category. These represented a significantly larger proportion of vaccine proponents (favor *n*= 116, 33%, oppose *n*=102, 29%, *X*^*2*^ = ^*39.56*^, p <0.001).

Thirteen percent shared political content, with 9% (*n*=175) of accounts posting conservative content and 4% (*n*=78) sharing liberal content. We defined “conservative” as tweeting in support of major Republican political figures (most commonly President Trump) or against major Democratic political figures or identities (e.g. disparaging “libs”). We defined “liberal” as the reverse. Accounts sharing conservative political content were more likely to also oppose vaccines (oppose *n=* 160, 91%, favor *n*=0, 0%). Although a similar number of accounts supporting vaccines (*n=*33) and opposing vaccines (*n*=26) also shared liberal politics content, this represented a significantly larger proportion of the vaccine proponents than vaccine opponents (7% vs 4%, *X*^*2*^ = 12.38, p =0.0004).

Nine percent (n=184) shared conspiracist content, split into general conspiracy theories (*n*=104, 5%) and conservative conspiracy theories (e.g. QAnon, New World Order, etc.) (*n*=82, 4%). By conspiracist, we are describing attempts to “explain events as secret acts of powerful, malevolent forces”(Jolley & Douglas, 2014). In this dataset this included allusions to shadowy groups like the Illuminati, complex theories that assume technology (e.g. 5G Wireless, “chemtrails,” etc.) is part of a plot to harm citizens, and more extreme theories proposing cover-ups, wrong-doing, and corruption within public health agencies, pharmaceutical companies, and charitable organizations. We made a distinction between conspiracy theories that were largely apolitical and did not explicitly name political figures, and those that were political, and frequently overlapped with conservative politics. Accounts tended to use distinctive hashtags representing both (e.g. using #QAnon or #WWG1WGA alongside #MAGA and #Trump2020). Recognizing that this distinction may be arbitrary, we also ran analysis using the combined conspiracy theory group. Almost all (96%, *n*=176) conspiracist content was shared by accounts also opposing vaccines.

Doctor/researcher accounts represented 6% (*n*= 114) and health organizations represented 5% (*n*= 102) of all accounts, with the majority (95%) of accounts in favor of vaccines. Both types of accounts tended to tweet about health issues more generally, which sometimes included vaccines.

Retweet counts for vaccine opponents (X^2^(7) =62.02 p<0.001) and proponents (X^2^(5)=64.12, p<0.001) both differed significantly by category using a non-parametric Kruskal-Wallis rank sum test. Among vaccine opponents, accounts that focused primarily on vaccination, and those focusing on conservative politics, had the most retweets -- both with a median of more than 100. In contrast, no accounts promoting vaccination achieved this degree of engagement. The most popular account category -- doctors/researchers -- achieved a median of 77 retweets and accounts focused primarily on vaccines were retweeted a median of 59.5 times.

After applying a logistic transform to control for floor and ceiling effects, bot scores also varied significantly by category among both vaccine proponents, *F(5, 469) = 15.25, p < 0.001*, and vaccine opponents, *F(7, 887) = 7.14, p < 0.001*, with news aggregators the most bot-like accounts for both opponents and proponents.

### Finding 3: The quality of COVID-19 information varied. The most reliable information included public health announcements and retweets of news, less reliable information came from discussion-oriented topics. Unreliable information included 5 distinct topics on conspiracy theories, 3 on rumors and insinuations, and 1 featuring scam “cures.”

We extracted 35 topics from COVID-19 related tweets generated by these accounts (more details provided in the Appendix). Only 5 of these 35 topics featured vaccines. We grouped these four categories: public health updates, news, discussion, and misinformation. These categories can also be thought of as more reliable information (public health updates and news topics), less reliable information and opinions (discussion topics), and unreliable information (misinformation topics).

Among the most reliable information topics, public health topics tended to feature direct retweets from health organizations (See Table 3, example: “Prevention Techniques”). News topics also tended to feature direct retweets of news headlines, often without additional commentary (example: “Deaths”). Most news topics focused on disease updates and the public health response, including emerging facts about the disease, continually updated case and death statistics, and news of vaccines in development.

Discussion topics featured fewer direct retweets and more personal comments, making these topics less reliable than news and public health topics. Several of these topics are very straightforward, with a clear theme that is consistent across most tweets (example: “Advocating Science”). Other discussion topics featured users agreeing on a general problem but disagreeing on specifics (example: “Infodemic”). The most debated topics were those that combined two or more similar arguments built around shared content (example: “Disease and Vaccine Narratives”). These claims were used both for and against vaccination, but the majority were used to downplay the severity of COVID-19. Many ended with appeals to vaccinate, but others took a different tack and decried “fear campaigns” from the media and “big pharma.”

Among the unreliable topics, we identified 5 topics on conspiracy theories, 3 based on rumors and insinuation, and 1 promoting scam cures. These conspiracy theory topics featured secret plots that connected powerful individuals and institutions to intentional harm. Some of these theories have already been widely covered in the mainstream media (example: “Chinese Bioweapon”) others were less widely circulated (example: “Engineered DNA”). The category of rumors/insinuation included topics that hinted at or suggested misdeeds, but often failed to commit fully to those claims. These topics were less disease focused and seemed to have more political agendas (example: “Chinese Coverup”). The single scam topic featured attempts to market natural cures.

### Finding 4: Anti-vaccine accounts were more likely to share unreliable information, including conspiracy theories about disease origins and criticism of China’s disease response. Pro-vaccine accounts shared more public health information, but also more discussion topics. Both types of accounts shared comparisons of disease severity, often downplaying the risks of COVID-19.

Assessing the distribution of topics among vaccine opponents we found approximately ⅓ of topics fell into each category: more reliable (30.1%), less reliable (34.5%), and unreliable (35.4%). In contrast, the distribution for vaccine proponents was roughly evenly split between more reliable (45.3%) and less reliable (43.4%), with only 11.3% unreliable topics. There are two important takeaways here: first, that vaccine opponents use a variety of content including both reliable and unreliable sources to make their arguments, and second, although it is less common, vaccine proponents are not exempt from spreading misinformation.

To focus on specific subgroups, we assessed the top three subgroups for both vaccine opponents and proponents. For vaccine opponents, the top three subgroups -- primarily vaccines, combined conspiracy theories, and conservative politics -- all presented similar topic breakdowns as the overall anti-vaccine topic model. Slight differences emerged: accounts tweeting primarily about vaccines and conspiracy theories shared a greater proportion of conspiracy topics than those tweeting about conservative politics. Accounts tweeting primarily about vaccines were also more clearly vaccine focused, featuring news of vaccine development and the greatest proportion of natural cures topics (6.4%). The vaccine-focused subgroup shared roughly the same proportion of conspiracy theory topics as the combined conspiracist subgroup (19.7% vs 19.8%). In addition to conspiracy theory topics, the combined conspiracy theories subgroup also incorporated reliable news (in the form of death rates and economic impacts) and discussion topics (both “disease narratives” downplaying severity, but also “coming pandemic” amplifying concerns).

Among vaccine proponents, doctors/researchers and health organizations were more closely aligned, featuring a greater proportion of reliable news and public health information. Health organizations were the only subgroup sharing a greater proportion of reliable news and public health topics than opinion topics and shared the lowest proportion of misinformation topics (7.5%). The accounts tweeting primarily about vaccines (not affiliated with doctors, researchers, or health organizations) shared a higher proportion of less reliable discussion topics (54.1%) including the largest share of the category “Disease & Vaccine Narratives.” We surmise that this subgroup was most active in spreading the “flu is worse so get a flu vaccine” type narratives within this topic.

## Methods

We utilized an archive of vaccine-related tweets (Dredze et al. 2014) to compile a list of the 2,000 most active vaccine-related accounts on Twitter. Accounts were included based on the total number of messages containing the keyword “vaccine” from January 1 to December 31, 2019. The most active account in our dataset shared 16,924 tweets meeting our inclusion criterion and the least active accounts shared 120 tweets. The distribution was skewed, with a max of 16,924 mentions, median of 218 mentions (IQR: 152.75–376.25), and minimum of 120 mentions.

We examined the screen name, Twitter handle, and if available, user-provided description for each account. Over a 12-day period in March 2020, two independent annotators (AJ, AS) manually assigned each account into one of 3 categories: pro-vaccine, anti-vaccine, or other. Annotators accessed each users’ Twitter page and public profile to assess recent content. Any accounts that were no longer available on Twitter, or had switched privacy settings to be private, were excluded from annotation.

After assessing vaccine sentiment, annotators noted what other types of content were shared by each account. While many accounts posted almost exclusively about vaccines, we also identified groups of accounts focusing on both liberal and conservative politics (e.g., expressing support for, or opposition to, specific political candidates or political parties), conservative conspiracy theories (e.g. QAnon, “Deep State,” etc.), general conspiracy theories (e.g. eugenics plots, opposition to 5G wireless, theories regarding mind control, chemtrails, etc.), natural/alternative health (e.g. veganism, chiropractors, naturopaths), doctors/researchers (e.g. “tweetatricians,” science journalists, epidemiologists), news accounts (including both original content and news aggregators), and a broad category of other accounts (e.g. joke bots, pet vaccines, music stations, general content) that fell under “mixed content.” If an account posted content from across multiple content areas or no discernable pattern was observed, it was also included as “mixed content.” Intercoder reliability was assessed with a random sample of 45 accounts, with 93% agreement on vaccine sentiment (Fleiss Kappa of 0.91, 0.81–1.00) and 78% agreement on subgroup assignment (Fleiss Kappa 0.75, CI: 0.61–0.89). Discrepancies in subgroup assignments were most common between accounts as doctor/researcher and health organization, conservative politics and conservative conspiracy theories, and in assignment of “mixed.” To address inconsistencies, annotators agreed that if no clear pattern emerged, “mixed” was the best choice.

Simultaneously, using Latent Dirichlet Allocation (LDA) (Blei et al., 2003), as implemented using the MALLET software package, we fit a topic model to 80,153 tweets collected from the 2,000 accounts (McCallum, 2002). These tweets all contained keywords related to COVID-19 including: ‘coronavirus’, ‘wuhan’, ‘2019ncov’, ‘sars’, ‘mers’, ‘2019-ncov’, ‘ncov’,’ wuflu’, ‘covid-19’,’ covid’, ‘#covid19’, ‘covid19’, ‘sars2’, ‘sarscov19’, ‘covid−-19’, ‘caronavirus’, ‘#trumpvirus’, ‘#pencedemic’, ‘#covid19us’, ‘#covid19usa’, ‘#trumpliesaboutcoronavirus’, ‘#pencepandemic.’ Using Bayesian hyperparameter optimization (Wallach et al., 2009), we de-rived 35 topics and generated 50 representative tweets from each topic. LDA Topic modelling assumes that words that commonly co-occur are likely to belong to a topic and that documents -- in this instance tweets -- that share common words are likely to share a similar topic. Each tweet receives a score that indicates how likely it is that the given tweet is related to each topic, higher scores reflect a higher degree of relatedness to the topic.

After reading the most representative tweets from each topic, the same annotators (AJ, AS) independently assigned a descriptive label to each topic. Labels were assessed as having high (nearly synonymous), moderate (significant overlap), or low agreement (little overlap). The majority (77%) were found to have high agreement; for instance, for topic 1, both annotators provided labels that were roughly synonymous: “How to protect yourself from coronavirus: washing hands, covering sneezes, etc.” and “How to protect yourself, and others, from the coronavirus.” In instances of moderate or low agreement, a third team member (DAB) was introduced and the team discursively addressed concerns until a unified topic was agreed upon. For instance, topic 6 was ultimately labelled “disease and vaccine”. It included both pro-vaccine sentiment (e.g. “Flu vaccines won’t prevent coronavirus but are still necessary”) and anti-vaccine sentiment (e.g. “a COVID-19 vaccine is unnecessary”) as well as general comparisons of disease severity between COVID-19 and other diseases. Annotators agreed on the broader label to capture the overlap between the multiple perspectives reflected in the topic. The two topics with low agreement were specific responses in non-US locales (Philippines and Hong Kong) and annotators may not have recognized place names.

Topics were also labelled into information categories. These included; “public health” meaning content was largely retweets from official public health sources; “news” meaning content was largely retweets of neutral news headlines (broadly-defined); “discussion” topics included more original content from users as well as retweets of commentary; and “misinformation” which included topics sharing conspiracy theories, unverified prevention and/or treatment options, as well as topics presenting unverifiable rumors or insinuations.

### Limitations

Topic models rely on probabilistic clustering algorithms to capture clusters of words that tend to co-occur in a given set of messages. Tweets that are the most representative of the underlying clusters are considered most relevant. By assessing the full-text versions of the most relevant tweets annotators were generally able to determine the prevailing sentiment of a topic (e.g. disseminating misinformation vs. debunking misinformation). However, especially for less relevant tweets, it is possible that tweets sharing similar keywords, but not similar sentiment were included but these tweets were less representative and therefore would have had minimal impact on analysis results. While this is important, given a dataset of this size it becomes less of a consideration.

We limited our analysis to English-language accounts and tweets but did not include any geographic bounds. A recent study over the same period found that while English language tweets accounted for only 34% of all tweets, they accounted for 58.7% of COVID-19 tweets (Singh et al., 2020).

## Figures and Tables

**Figure 1.**
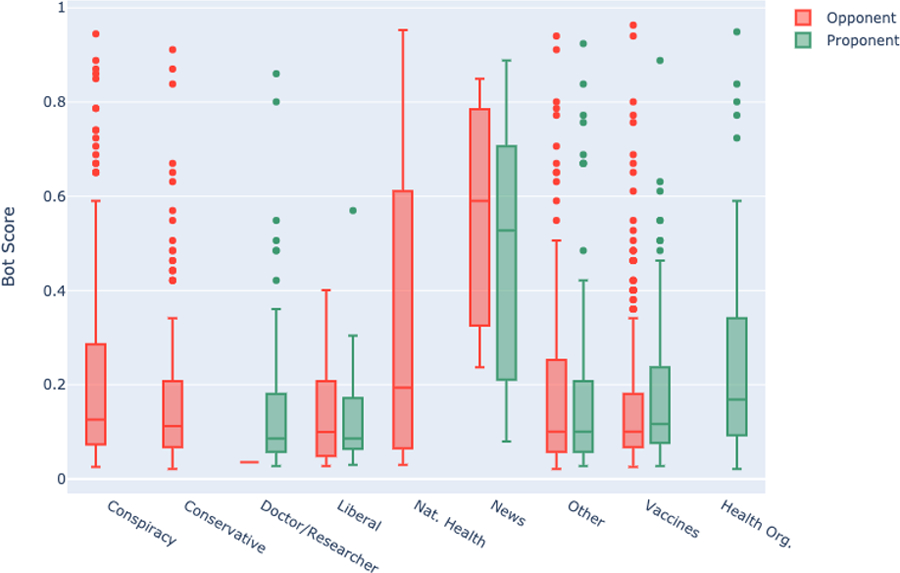
Retweets by subgroup. Boxplots show distribution of retweets for each subgroup, dots represent outliers. For an interactive version, please visit: https://broniatowski.github.io/Not-Just-Conspiracy-Theories/Figure%201.html

**Figure 2.**
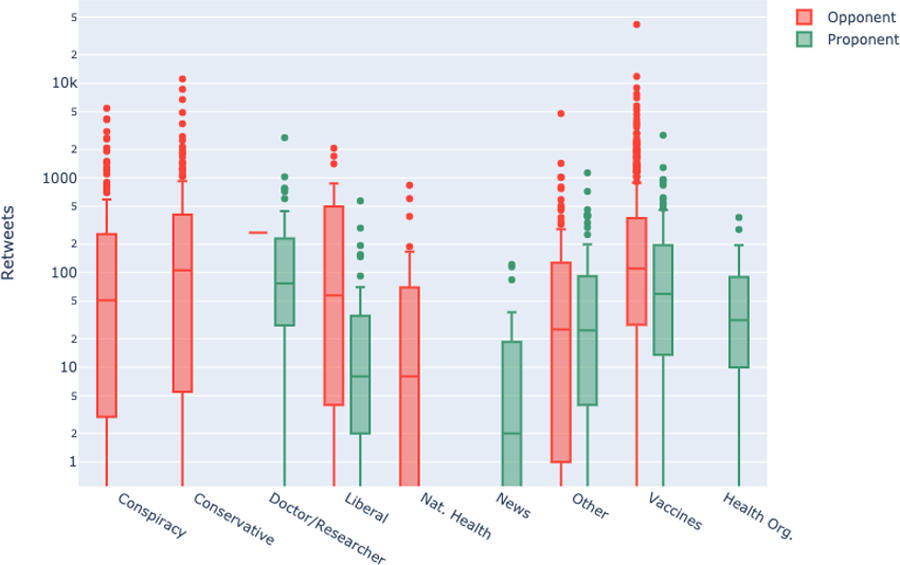
Botometer scores by sub community. Boxplots represent distributions of scores, with dots representing outliers. Scores > 0.43 were considered likely bots. Only news accounts appeared to have a significant proportion of likely bots. For an interactive version, please visit: https://broniatowski.github.io/Not-Just-Conspiracy-Theories/Figure%202.html

**Figure 3.**
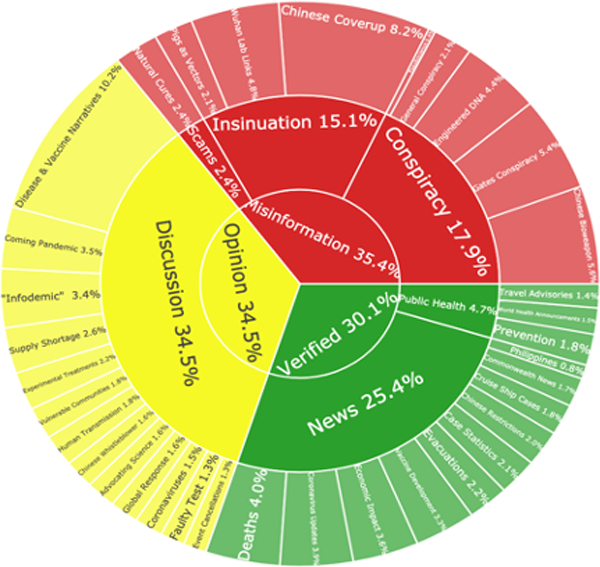

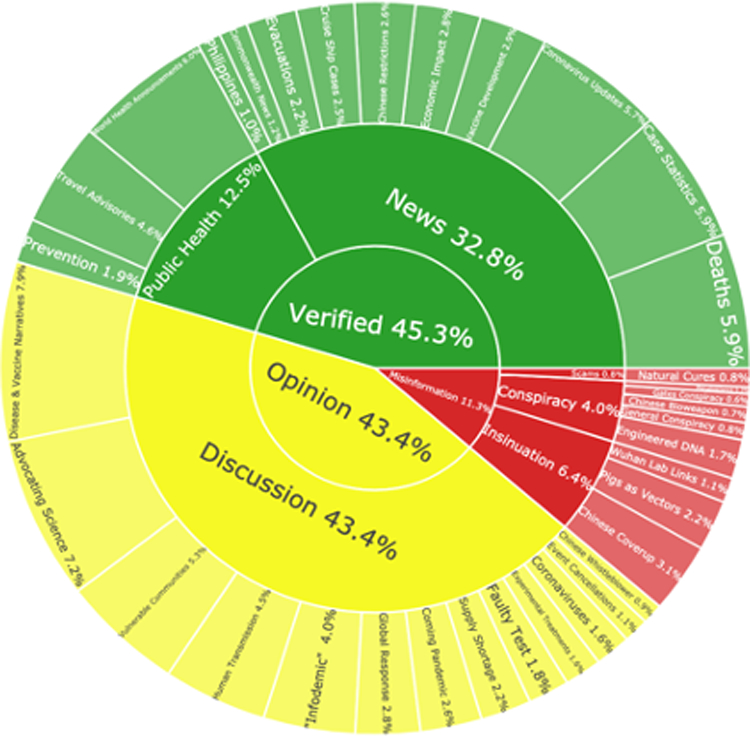
Breakdown of topics for both vaccine opponents (top) and vaccine proponents (bottom). Colors: red = unreliable, yellow = less reliable, green = more reliable. For an interactive version of the top figure, please see: https://broniatowski.github.io/Not-Just-Conspiracy-Theories/Figure%203a.html. For an interactive version of the bottom figure, please see: https://broniatowski.github.io/Not-Just-Conspiracy-Theories/Figure%203b.html

**Table 1.** Breakdown of top vaccine accounts on Twitter.

Vaccine Opponents (n*=*905)	Vaccine Proponents (n*=*479)	Other (n*=*305)
Primarily Vaccines 398 (44%) Combined Conspiracies 176 (19%) General 96 Conservative 80 Conservative Politics 160 (18%) Mixed Content 102 (11%) Natural Health 39 (4%) Liberal Politics 26 (3%) News Accounts 3 (>1%) Doctor/Researcher 1 (>1%)	Mixed Content 116 (24%) Doctor/Researcher 109 (23%) Primarily Vaccines 100 (21%) Health Organization 94 (20%) Liberal Politics 33 (7%) News Account 27 (5%)	Mixed Content 129 (43%) News Account 64 (21%) Foreign Language 39 (13%) Conservative Politics 15 (5%) Music 14 (5%) Pets 13 (4%) Liberal Politics 9 (3%) Combined Conspiracies 8 (3%) General 6 Conservative 2 Natural Health 8 (3%) Doctor/Researcher 4 (1%) Health Organization 2 (<1%)

**Table 2.** Topics from LDA analysis by type.

**Most Reliable**	*Public Health Topics (3)*: Prevention Techniques*, WHO* Announcements, Travel Warnings *News Topics (11):* Coronavirus Updates, Case Statistics, Deaths, Cruise Ship Cases, Evacuations*, Vaccine* Development, Economic Disruption, Event Cancellations, Philippine News, Commonwealth News, Chinese Restrictions
**Less Reliable**	*Discussion Topics (12):* Disease & Vaccine Narratives, Human Transmission, Coronaviruses, Faulty Tests, Advocating for Science, Medical Supply Shortages, Comparing Global Responses, Experimental Treatments, *Infodemic*, Social Impacts, Chinese Whistleblower, Coming Pandemic
**Unreliable**	*Conspiracy Theories (5):* Gates Foundation Conspiracy, Chinese Bioweapon, DNA Conspiracy, Pseudoscience, General Conspiracy *Insinuation/Rumors (3):* Links to Wuhan Lab, Pigs as Vectors, Chinese Coverup, *Scams (1):* Natural Cures

**Table 3.** Examples of topics with representative Tweets.

Topic	Description	Excerpts from representative tweets
Public Health: Prevention Techniques	Retweets of prevention methods from the WHO and other public health agencies	#RT @WHO: Protect others from getting sick: Avoid close contact and wash hands when you are experiencing fever and cold or flu-like symptoms 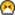 . Seek medical care 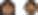 if you have a fever 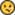 , cough and difficulty breathing. #coronavirus
News: Deaths	Retweets of news featuring updated death tolls	Novel coronavirus deaths spike again as outbreak shows no signs of slowing… the number of cases in China grew by 3,694, or 15%, on the previous day. There have been 563 deaths
Discussion: Disease & Vaccine Narratives	Comparisons of disease susceptibility & severity -- most often with influenza.	● Context: there are no coronavirus deaths in america. 10,000 influenza deaths this year... perspective not panic please. ● If you live in Wuhan, be concerned. If you live in the us, go get a #flushot. ● previous fear campaigns 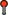 2020 corona virus 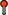 2016 zika 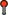 2014 ebola 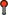 2009 swine flu 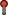 ● With wall-to-wall news coverage, it’s natural to worry about the coronavirus, but the #flu is currently far more dangerous here in the us... it’s not too late to get a #flushot 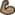
Discussion: Advocating Science	Describing need for evidence-based response and “facts not fear”	● We need to: 1 work together 2 ensure that we have all necessary resources 3 bring our best science to the forefront ● In a disease outbreak it’s important to focus on “facts, not fear.”
Discussion: Infodemic	Describing what the infodemic is and why it is a problem.	● @who: @drtedros “At WHO, we’re not just battling the #2019ncov virus; we’re also battling the trolls and conspiracy theorists that push misinformation and undermine the outbreak response… ● Misleading, unverified and false information about the coronavirus has spread across social media platforms...
Conspiracy Theory: Chinese Bioweapon	Theories that the virus was intentionally created as a bioweapon.	● 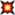 In explosive interview, author of bioweapons act confirms coronavirus is an “offensive biological warfare weapon” ● Breaking: coronavirus a race-specific bioweapon that’s breaking containment worldwide
Conspiracy Theory: Engineered DNA	Theories that genetic analysis reveals the virus was lab developed.	‘Study of the coronavirus genetics shows this new found strain may have been engineered and “accidentally” released for vaccine creation.’
Rumor/Insinuation: Chinese Coverup	Suggestions that China is covering up the true death toll and using extreme measures to keep the population under control.	● Cremation vans running 24/7 as China orders door-to-door mass roundups of infected citizens... ● I hope this is not true… Chinese police shoot and kill the suspected infected that refuse to be evacuated
Scam: Natural Cures	Advertising products (or links to further information on products) as cures for COVID.	● The body makes a cure for cancer & coronavirus: anti neo plastrons found in our powerful pee. Drink all of your perfect pee daily & you are protected. - Call 877-XXX-XXX. no vax. needed. ● Vitamin C clinical trial for coronavirus in china. people who take 2 gm. Vit. C hourly do not get coronavirus or autism.

**Table 4.** Breakdown of topics for top 3 subgroups (featuring topics >4%).

	More Reliable	Less Reliable	Unreliable
**Vaccine Opponents** ***905 Accounts***	**30.1%**	**34.5%**	**35.4%**
Primarily Vaccines *398 Accounts*	**19.6%** Vaccine Development 6.3%	**36.8%** Disease Narratives 13.4%	**38.3%** Gates Conspiracy 6.8% Natural Cures 6.4% Engineered DNA 6.2% Chinese Coverup 5.1% Wuhan Lab Links 5.0% Chinese Bioweapon 4.6%
Combined Conspiracy Theories *176 Accounts*	**26.2%** Deaths 4.7% Economic Impact 4.0% Virus Updates 4.0%	**32.6%** Disease Narratives 8.3% Coming Pandemic 4.9%	**36.7%** Chinese Coverup 9.3% Chinese Bioweapon 7.1% Gates Conspiracy 5.5% Wuhan Lab Links 4.8% Engineered DNA 4.4%
Conservative Politics *160 Accounts*	**28.3%** Economic Impact 5.1% Deaths 4.4% Virus Updates 4.2%	**33.0%** Disease Narratives 8.7%	**34.4%** Chinese Coverup 9.5% Chinese Bioweapon 5.7% Gates Conspiracy 5.0% Wuhan Lab Links 4.8%
**Vaccine Proponents** ***479 Accounts***	**45.3%**	**43.4%**	**11.3%**
Doctor/Researcher *109 Accounts*	**43.1%** Case Statistics 6.8% Deaths 6.3% Virus Updates 5.8% WHO Announcements 4.6%	**44.9%** Disease Narratives 9.5% Advocating Science 7.8% Human Transmission 5.5% Vulnerable Communities 5.2%	**12%**
Primarily Vaccines *100 Accounts*	**34.6%** Vaccine Development 8.7% Virus Updates 4.2%	**54.1%** Disease Narratives 16.9% Infodemic 6.9% Advocating Science 5.0% Transmission 4.9% Vulnerable Communities 4.4%	**11.2%**
Health Organizations *94 Accounts*	**47.3%** WHO Announcements 13.8% Travel Advisories 8.9% Case Statistics 5.3% Vaccine Development 4.2% Virus Updates 4.0%	**45.2%** Advocating Science 12.7% Coronaviruses 10.1% Infodemic 4.8% Transmission 4.1%	**7.5%**

## Research questions

- How did existing online communities of vaccine opponents and proponents respond to early news of the novel coronavirus?
- What were the dominant topics of conversation related to COVID-19? What types of misleading or false information were being shared?
- Which types of accounts shared misleading or false content? How do these topics vary between and within vaccine opponent and vaccine proponent Twitter communities?
- What do these findings mean for efforts to limit online health misinformation?

## Essay summary

- We identified the 2,000 most active Twitter accounts in the vaccine discourse from 2019, identifying both vaccine opponents and proponents. Only 17% of this sample appeared to be bots. In addition to tweeting about vaccines, vaccine opponents also tweeted about conservative politics and conspiracy theories. Vaccine proponents tended to represent doctors, researchers, or health organizations, but also included non-medical accounts.
- On February 20, we collected the most recent tweets for each account and automatically extracted 35 distinct topics of conversation related to COVID-19 (roughly 80,000 tweets). Topics were categorized as: more reliable (public health updates & news), less reliable (discussion), and unreliable (misinformation). Misinformation included conspiracy theories, unverifiable rumors, and scams promoting untested prevention/cures.
- Vaccine opponents shared the greatest proportion (35.4%) of unreliable information topics including a mix of conspiracy theories, rumors, and scams. Vaccine proponents shared a much lower proportion of unreliable information topics (11.3%).
- Across both vaccine proponents and vaccine opponents, the largest single topic of conversation was “Disease & Vaccine Narratives,” a discussion-based topic where users made comparisons between COVID-19 and other diseases -- most notably influenza. These messages likely added to public confusion around the seriousness and nature of COVID-19 that endures months later.
- In the context of an ‘infodemic,’ efforts to address and correct misinformation are complicated by the high levels of scientific uncertainty. Focusing on only the most conspicuous forms of misinformation -- blatant conspiracy theories, bot-driven narratives, and known communities linked by conspiracist ideologies -- is one approach to addressing misinformation, but given the complexity of the current moment, this strategy may fail to address the more subtle types of falsehoods that may be shared more broadly.

## Appendix

**Table 5.** Topics inferred using LDA

#	Label	Category	Keywords
1	Prevention techniques	Public Health	coronavirus hands amp wash protect sick avoid water house ncov virus contact masks cough hand scientists cotton coughing asks don’t
2	Gates Foundation conspiracy theory	Misinformation -- Conspiracy Theory	coronavirus gates bill amp patent pandemic outbreak simulation million **vaccine** foundation institute melinda predicted months pirbright world wuhan ago kill
3	Coronavirus updates	News	coronavirus case confirmed wuhan patient china health hospital positive tested cases infected patients people chinese virus person ncov man diagnosed
4	Human transmission	Discussion	coronavirus ncov transmission wuhan china cases spread symptoms study patients infected pneumonia covid human infection evidence outbreak asymptomatic human-to-human period
5	WHO announcements	Public Health	coronavirus health emergency ncov outbreak public global amp international drtedros committee world covid response declares whoafro declared countries whoemro whosearo
6	Disease & vaccine narratives	Discussion	coronavirus flu people sars virus it’s amp wuhan don’t time **vaccine** ncov covid it’s fear bad china pandemic don’t i’m
7	Links to Wuhan lab	Misinformation -- Rumor/Insinuation	coronavirus wuhan lab virus chinese china scientists originated scientist laboratory outbreak period incubation pathogens expert research level university days aren’t
8	Evacuations	News	wuhan coronavirus china quarantine flight citizens passengers americans outbreak u.s military evacuees evacuated base evacuation air home airport california nationals
9	Infodemic	Discussion	coronavirus information ncov misinformation outbreak covid twitter news conspiracy spread wuhan latest media amp china times government theories social people
10	Case statistics	News	cases coronavirus confirmed china ncov case reported total deaths covid wuhan update reports infection korea health report suspected jan south
11	Chinese Whistleblower	Discussion	coronavirus doctor wuhan chinese wenliang journalist citizen alarm early dies died cdc death whistleblower beckyjohnson police life rosewind mcfunny california
12	Vaccine development	News	coronavirus **vaccine** develop **vaccines** ncov development make research company biotech amp working pharma race virus mers covid work prevention outbreak
13	Chinese coverup	Misinformation--Rumor/Insinuation	coronavirus wuhan china hospital people quarantine chinese outbreak city video patients million infected lockdown hospitals bodies dead days police inside
14	Faulty tests	Discussion	coronavirus test kits ncov testing tests cdc china amp covid countries negative diagnostic false virus results africa labs health emergency
15	Deaths	News	coronavirus china cases death toll deaths outbreak reports hubei confirmed updates number people virus province live reported infections wuhan latest
16	Natural cures	Misinformation -- alternative cures	coronavirus amp paul cure pee kangas youtube hourly daily **vaccines** vitamin vit drink body protected makes found perfect grams china
17	Cruise ship cases	News	ship cruise coronavirus japan passengers positive princess diamond quarantined cases people covid tested quarantine test americans aboard confirmed news board
18	Travel warnings	Public Health	coronavirus ncov china wuhan outbreak travel virus cdc health disease risk infection states control spread screening guidance united covid u.s
19	Economic disruption	News	coronavirus china outbreak impact global economy business hit february article economic supply due warns chinese markets scoopit apple market amid
20	Advocating for science	Discussion	ncov coronavirus amp covid outbreak china drtedros cdc health drnancym_cdc data secazar information drmikeryan response working public countries spread time
21	Philippine news	News	ncov coronavirus covid doh travel filipinos philippines health amid department ban measures city due secretary government patients duterte task disease
22	Experimental treatments	Discussion	coronavirus drug hiv patients treatment drugs china clinical doctors trials trial ncov antiviral news treat vitamin fight chinese wuhan plasma
23	Pigs as vectors	Misinformation -- rumors/insinuati ons	wuhan coronavirus pigs pneumonia china pork virus market source outbreak infected scientists chinese people seafood epidemic swine sars coming animal
24	Chinese bioweapon	Misinformation -- Conspiracy	coronavirus biological china weapon chinese outbreak wuhan bioweapons youtube warfare act truth bioweapon infowars interview boyle francis video amp expert
25	Coming pandemic	Discussion	coronavirus spread pandemic china warns global covid experts cdc coronavirusoutbreak virus world prepare wuhan u.s military warn ncov official health
26	DNA conspiracy	Misinformation-- Conspiracy	coronavirus ncov virus hiv bat scientists covid sars engineered genetic lab spike bioweapon found protein wuhan created pheic viruses biosecurity
27	Medical supply shortage	Discussion	coronavirus masks medical workers wuhan face china mask covid supplies outbreak infected doctors protective people health amid staff wearing shortage
28	Commonwealth news	News	news coronavirus media canada published outbreak global scoopit abc latest covid web social ecosearch daily bbc skinnergj fake australian feedly
29	Pseudoscience	Misinformation-- Conspiracy	wuhan coronavirus crisis pathology viral biophysically linked link constrained latency virus-driven heart jordivisiond esoxcarcharias maryvmos loriniowa volumerose corde cant_read_maps monsta
30	Comparing global responses	Discussion	health coronavirus world stigma china public outbreak organization india minister covid officials china’s medical vigorbot global nigeria fight u.s government
31	General conspiracy	Misinformation-- conspiracy	coronavirus infect population big deaths pharma rate amp china **vaccine** wuhan wall caution covid agenda horse top don’t urges kong
32	Chinese restrictions	News	coronavirus kong hong china travel outbreak flights chinese border amid spread ban wuhan due airlines australia restrictions government borders city
33	Event cancellations	News	coronavirus theresistance impeachtrump due thamendmentnow fears outbreak amid year china event show postponed world covid concerns chinese events epidemic congress
34	Coronaviruses	Discussion	mers sars coronavirus respiratory disease virus syndrome ncov amp east saudi severe viruses flu middle cases wuhan covid ebola mers-cov
35	Social impacts (e.g. stigma, bullying)	Discussion	coronavirus children china stigma outbreak chinese wuhan home people school fears stay schools parents amid kids students work family china’s
